# A simplified GIS and google-earth-based approach for lineaments and terrain attributes mapping in a basement complex terrain

**DOI:** 10.1038/s41598-022-20057-2

**Published:** 2022-09-22

**Authors:** M. A. Lawal, A. O. Oshomoji, A. A. Akinlalu, K. O. Omosanya, O. S. Ndukwe, K. A. N. Adiat, G. O. Mosuro

**Affiliations:** 1 Dr. Moses Strauss Department of Marine Geosciences, University of Haifa, Haifa, Israel; 2 Department of Geosciences, University of Lagos, Akoka, Nigeria; 3 Department of Applied Geophysics, Federal University of Technology, Akure, Nigeria; 4 Oasisgeokonsult, 7052 Trondheim, Norway; 5 Department of Geology, Federal University of Oye-Ekiti, Oye-Ekiti, Nigeria; 6 Department of Earth Sciences, Olabisi Onabanjo University, Ago-Iwoye, Nigeria

**Keywords:** Structural geology, Geology, Geomorphology, Hydrogeology

## Abstract

In this study, we use an integrated geologic mapping technique for remote mapping of lineaments and geologic terrain. Our workflow is based on geographic information system tools and consists of stream network delineation, lineaments mapping, terrain roughness index calculation, and interpretation of structural fabrics from google earth aerial photographs. The case study area, the Idanre Hills in southwestern Nigeria, has a protracted history and is characterized by steep-sided outcrops of a granitic batholith and migmatite-gneiss. Lineaments are widespread and dense around the batholith, occurring in areas of high elevation, and slope gradient. Terrain roughness indices are high at the outcrops and lineament sites. Streams in the area exhibit variable flow and partly align with the lineaments. The high roughness indices observed have tectonic connotations and are related to the occurrence of lineaments, strain domains, and high degree of rock weathering. Importantly, our method is effective in remote mapping of lineaments and terrain attributes within the study area and has wider applications in other basement complex terrains.

## Introduction

The availability of digital elevation models (DEMs) and digital surface models (DSMs) of almost all places on earth through platforms such as Google Earth, USGS earth explorer, and NASA’s world wind, and recent advances in geospatial technologies have enabled remote study of the earth’s geology and terrain properties^1–5^. These DEMs come with improved ability to perform geospatial analysis^3^ using tools such as ArcGIS and QGIS, and to virtually delineate geological features such as lineaments on a local to regional scale^6,7^. Lineaments are mappable linear geological features on earth’s surface and include faults, joints, shear zones, intrusions, and dykes. They are usually developed in areas of high stress or strain concentration following rifting or orogeny^8,9^.

Lineaments can be mapped through conventional geological field mapping^10^, which is generally laborious, costly and time-consuming, and via manual or automatic extraction from topographical maps, DEMs^11^, geological maps^12^, aeromagnetic data^13^, seismic reflection data^14,15^, and satellite images^11,16,17^. Moreover, integration of spatial data and geographic information system (GIS) techniques^2,18^ has aided the study of terrain properties such as slope, hillshade^18^, and roughness^19^. These properties are crucial for geomorphological studies of the earth’s topography. Of greater significance is the integration of spatial data and GIS techniques for the analysis of topographic expressions such as lineaments, which are important for understanding tectonic evolution of the earth^7,20^.

Understanding the occurrence and impact of lineaments is vital in groundwater and ore mineral exploration as lineaments are favorable zones for groundwater migration and storage^21,22^, hydrothermal fluid migration, and ore mineral emplacement^23,24^. Since seismic hazards commonly occur in or near zones of intense and active faulting or tectonic movements^25,26^, knowledge of the occurrence and behavior of lineaments may be important in choosing building, dams, and bridge construction sites^27^. Lineaments such as faults may function as migration pathways for hydrocarbon (and other fluids) in the subsurface^28,29^, and are therefore commonly considered during hydrocarbon exploration^30^. Fracture porosity, which increases in high fracture density zones, is also important for enhanced oil and gas recovery^31^. Today, knowledge of the occurrence and behavior of fractures is important in the assessment of subsurface geological units for their storage potentials e.g., CO_2_^32^.

**Figure 1:**
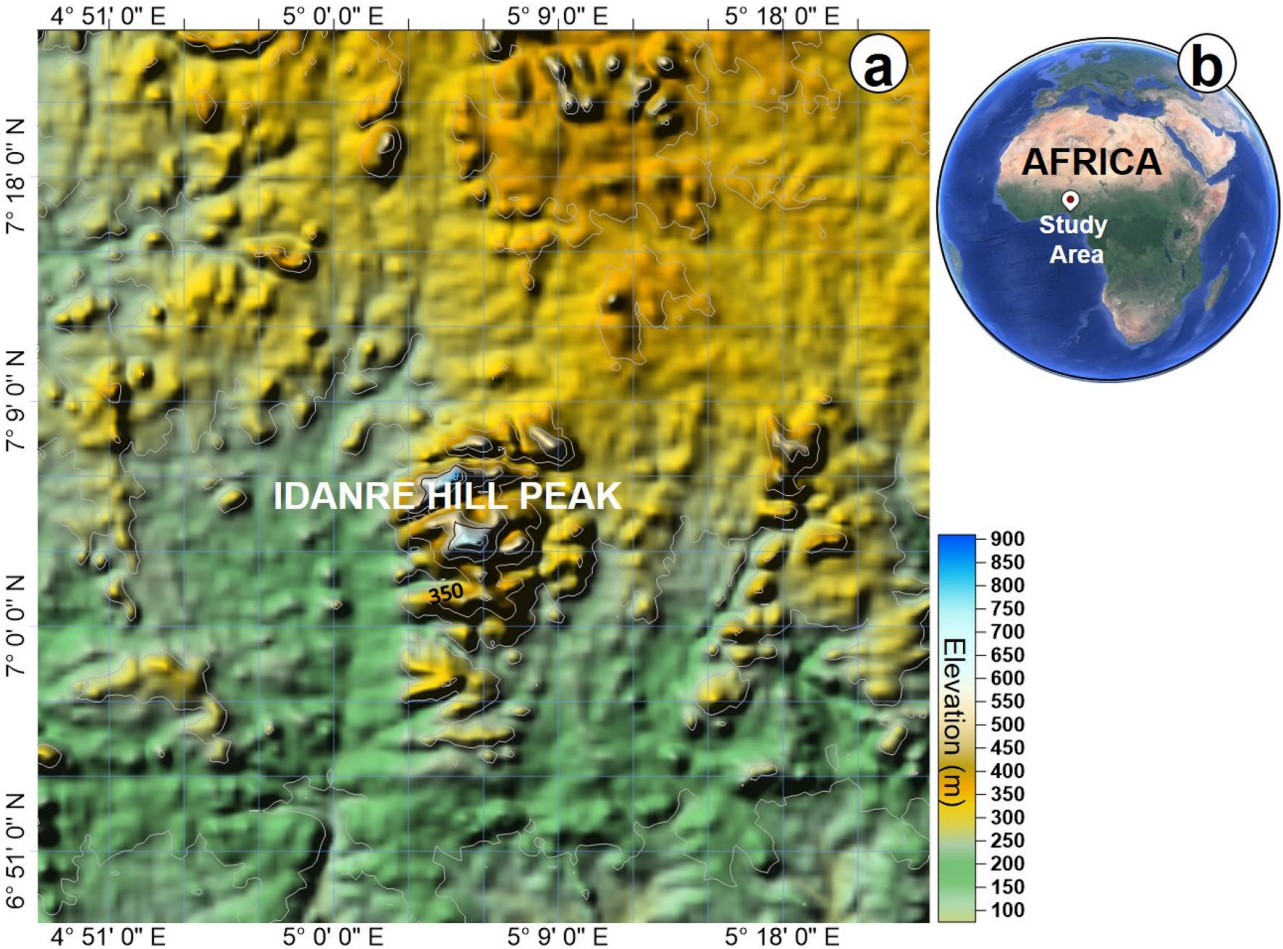
Location map of the study area. (**a**) Topographic map of the study area downloaded from Topex global topography data (https://topex.ucsd.edu/cgi-bin/get_data.cgi) and (**b**) Inset map showing location of the study area in the context of Africa.

**Figure 2:**
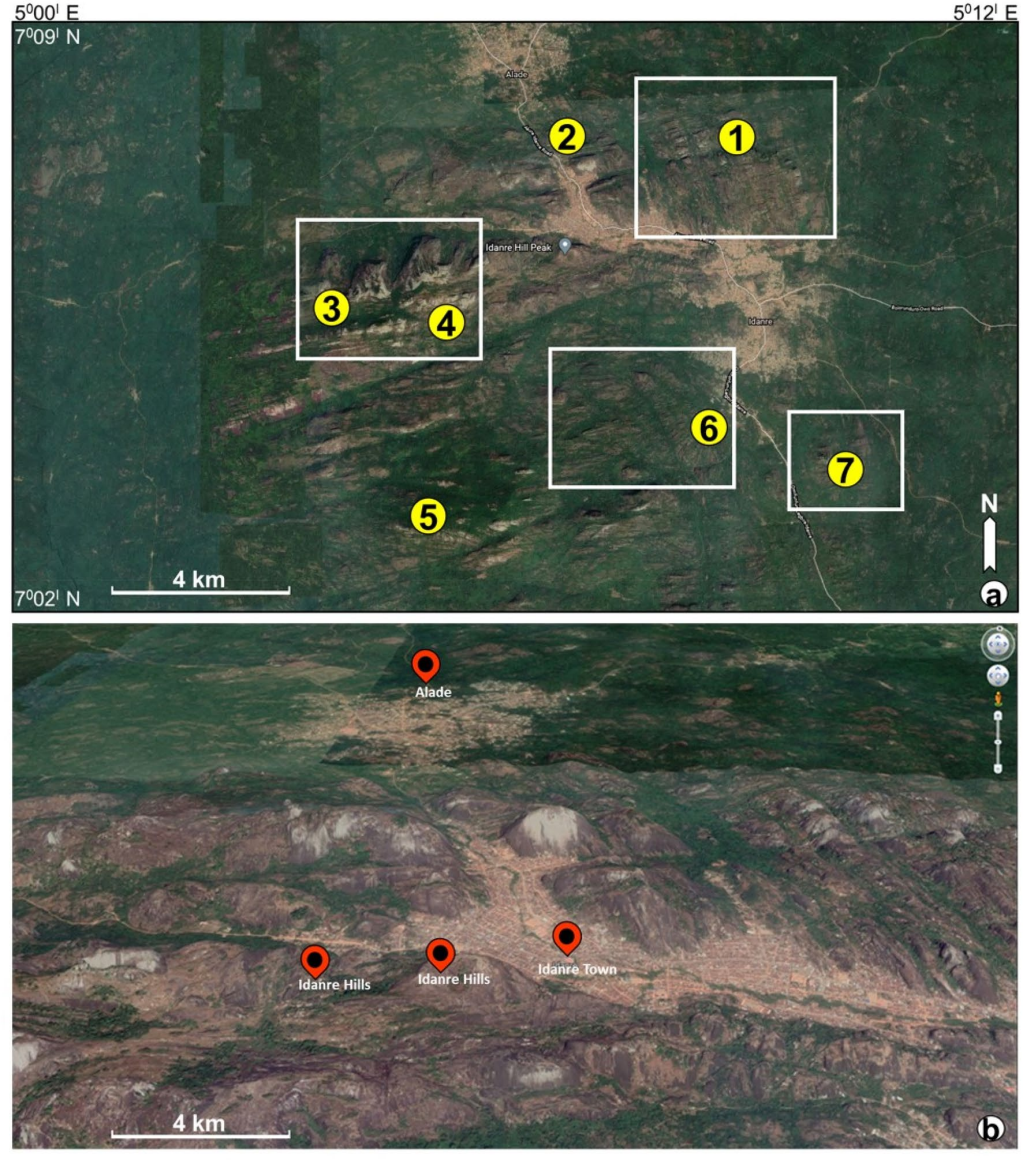
(**a**) Location map of the study as downloaded from google earth. Points marked as 1–7 highlight areas with interesting geological features and structures. (**b**) 3D view through the Idanre Hill and surrounding structural features. Images/Maps data: Google Earth, Maxar Technologies and CNES/Airbus.

Recognition and mapping of lineaments on open source and readily available data such as satellite or google earth images^6,7,33^ are important in large sites that are partially accessible for conventional field mapping or better still for reconnaissance prior to detailed geologic field mapping. However, in the case of google earth imagery, despite its meter- to cm-scale spatial resolution, which permits visualization of features and the wide variety of information obtained from it, it is generally undervalued for geological studies. In this study, we apply an integrated workflow to reveal the suitability of GIS techniques and google earth imagery for remote mapping of lineaments and terrain characterization within a basement complex terrain. To achieve this aim, we studied a natural laboratory, the Idanre Hills in southwestern Nigeria. The Idanre Hills is covered by satellite data and characterized by highly deformed rock outcrops (Figs. 1 and 2). These rocks extend several tens of kms within the basement complex terrain of Nigeria. The basement complex of Nigeria represents an assemblage of Precambrian migmatite-gneiss-quartzite, schist and Older granite rocks^34,35^.

**Figure 3:**
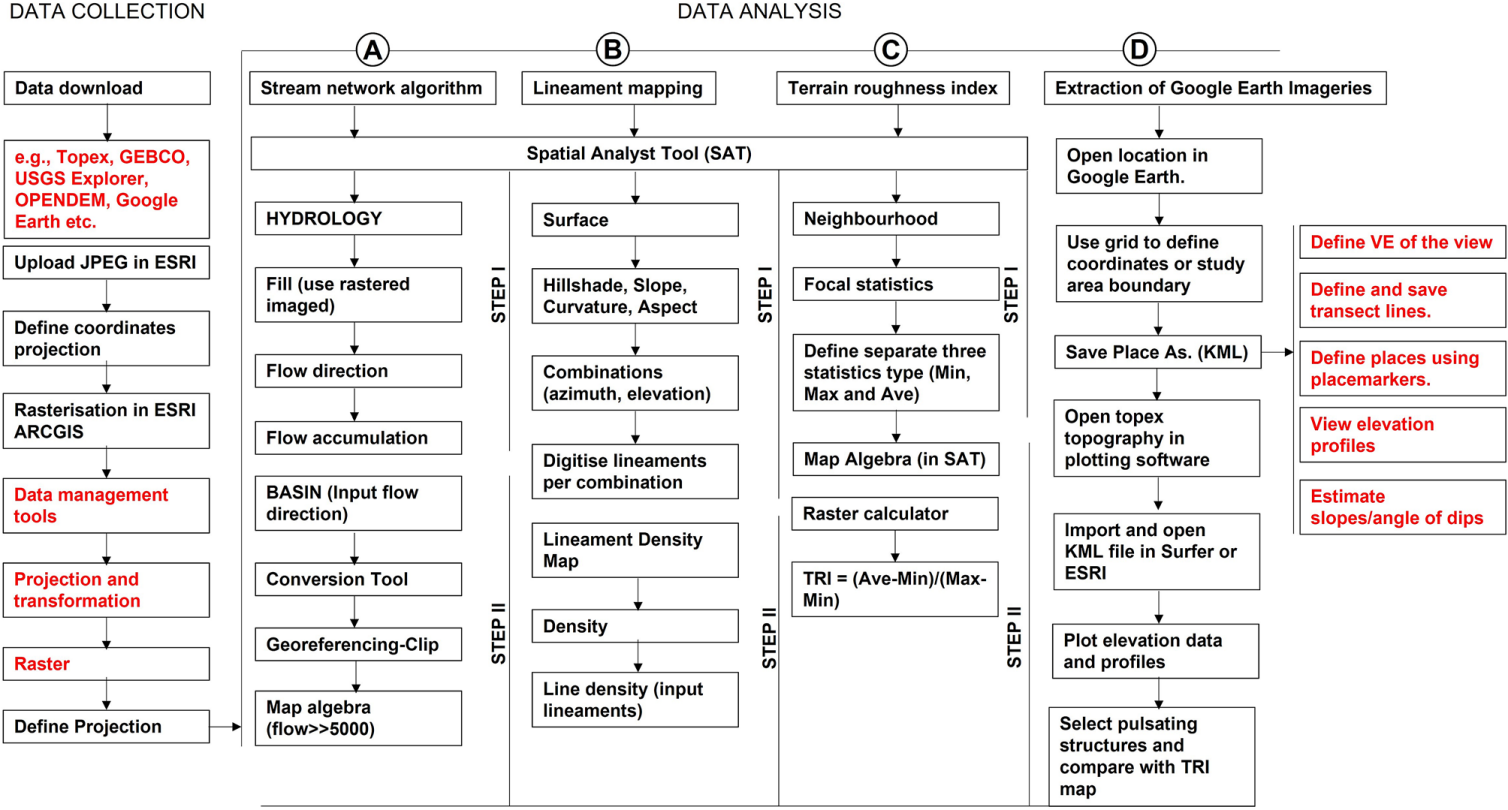
Workflow and algorithms used in this study. The workflow is divided into two main parts; Data collection and Data analysis, including (**a**) stream network delineation (**b**) lineament mapping (**c**) terrain roughness index calculation, and (**d**) extraction of google earth images. N.B: sub-processes are shown in red font.

Integrated use of GIS techniques and google earth aerial photographs for lineament mapping and terrain studies within the Nigerian basement complex is unknown or poorly documented to date. Few previous studies used remote sensing data to investigate the emplacement of the Idanre batholith^36^, highlight brittle and ductile deformation in rocks^37^, assess impact of lineaments and geology on groundwater potential^17,38,39^, studied the nature of lineaments and lithology^40^, and evaluated structural features within the Ife-Ilesha schist belt^41^ in parts of the Nigerian basement complex. In this current study, lineaments were extracted from multi-directional hillshade maps generated from google earth DSMs of the Idanre Hills (Fig. 3). These maps have the advantage of producing unprecedented images of geological features by computing hillshade from different directions as opposed to the commonly used hillshade that is computed in one direction^8,21^. Terrain attributes were also studied by generating slope, elevation, and terrain rough index maps, as well as stream network of the study area (Fig. 3). Our approach proved to be effective in extracting 143 lineaments and for characterizing several disparate terrain attributes. The methods and results from this study have overarching applications for remotely mapping geology, in reconnaissance survey, and in characterizing structures and terrain types in basement complex environments.

## Location and geology of the study area

The Idanre Hills, the study area, lie within latitudes N 7° 02′–N 7° 08′ and Longitudes E 5° 00′–E 5° 12′ and has an elevation of 100 m to 900 m above sea level (Figs. 1 and 2). It is part of the basement complex terrain of Nigeria, which lies between the West African and Congo cratons, south of the Tuareg Shield, and forms part of the Pan-African mobile belt^42–44^. The Nigerian basement complex evolved through four orogenic phases, including the Liberian (2700 Ma), the Eburnean (2000 Ma), the Kibaran (1100 Ma), and the Pan African (± 600 Ma) orogenies^45^. The Liberian, Eburnean, and Kibaran orogenic phases were associated with intense deformation, isoclinal folding, regional metamorphism, and extensive migmatization. The last orogenic event i.e., the Pan-African orogeny is a regional tectono-thermal event that involved collision-related orogenic activities^46^. The Pan-African orogeny was characterized by granitization and gneissification, which produced syntectonic granites, homogeneous gneisses, regional metamorphism, and migmatization. Importantly, the Pan African orogeny caused high-level structural overprinting and re-calibration of several geochronological clocks in older rocks^47,48^, evidences of which are preserved in several outcrops within the Nigerian basement complex.

Rock types found within the basement complex of Nigeria can be classified into 3 main groups: (1) the migmatite-gneiss-quartzite complex, (2) the schist belts, and (3) the Older granite suite^48^. The migmatite-gneisses are foliated and are the oldest rock types within the basement complex on a regional scale. The schist belts are low-grade sediment-dominated NNE-SSW trending rocks that are commonly in-folded into the migmatite–gneiss–quartzite complex^49^, while the older granites are commonly found within older rocks as high-level intrusions and rocks such as tonalites, diorites, granodiorites, granites, syenites, and charnockites^48,50,51^. Rock types found within the Idanre Hills area include Older porphyritic granites, migmatite-gneiss, Neoproterozoic metasediments^52^, and charnockites^51,53^. The Older granites are calc-alkaline rocks^53^ and are often classified as pre-, syn- and post-tectonic rocks that are associated with the Pan-African orogeny. They were emplaced concordantly to semi-concordantly within the foliated migmatite-gneiss and in the overlying metasedimentary cover as a batholithic mass, often referred to as the Idanre batholith^36,39^. In terms of field observations, several lineaments and xenoliths of the migmatite-gneiss have been identified within the Older granites^36^. This is in addition to multiple weathering-derived coarse boulders of the granites^36,53^.

## Streams, lineaments and terrain characterization in the study area

### Stream network and topography

**Figure 4:**
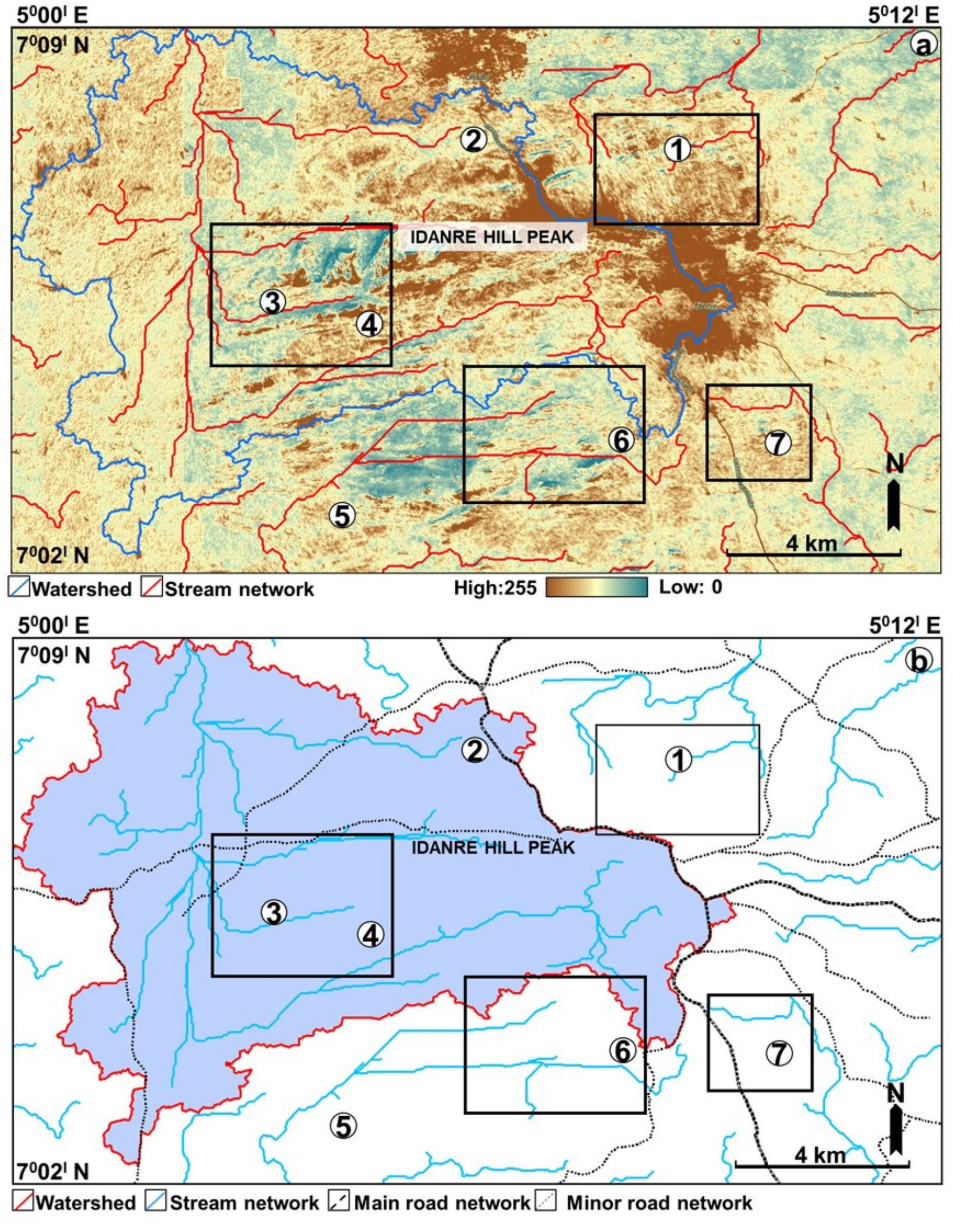
(**a**) Elevation of the study area overlain by stream network derived from the hydrology algorithm (**b**) Stream network map showing the outline of the mapped watershed.

**Figure 5:**
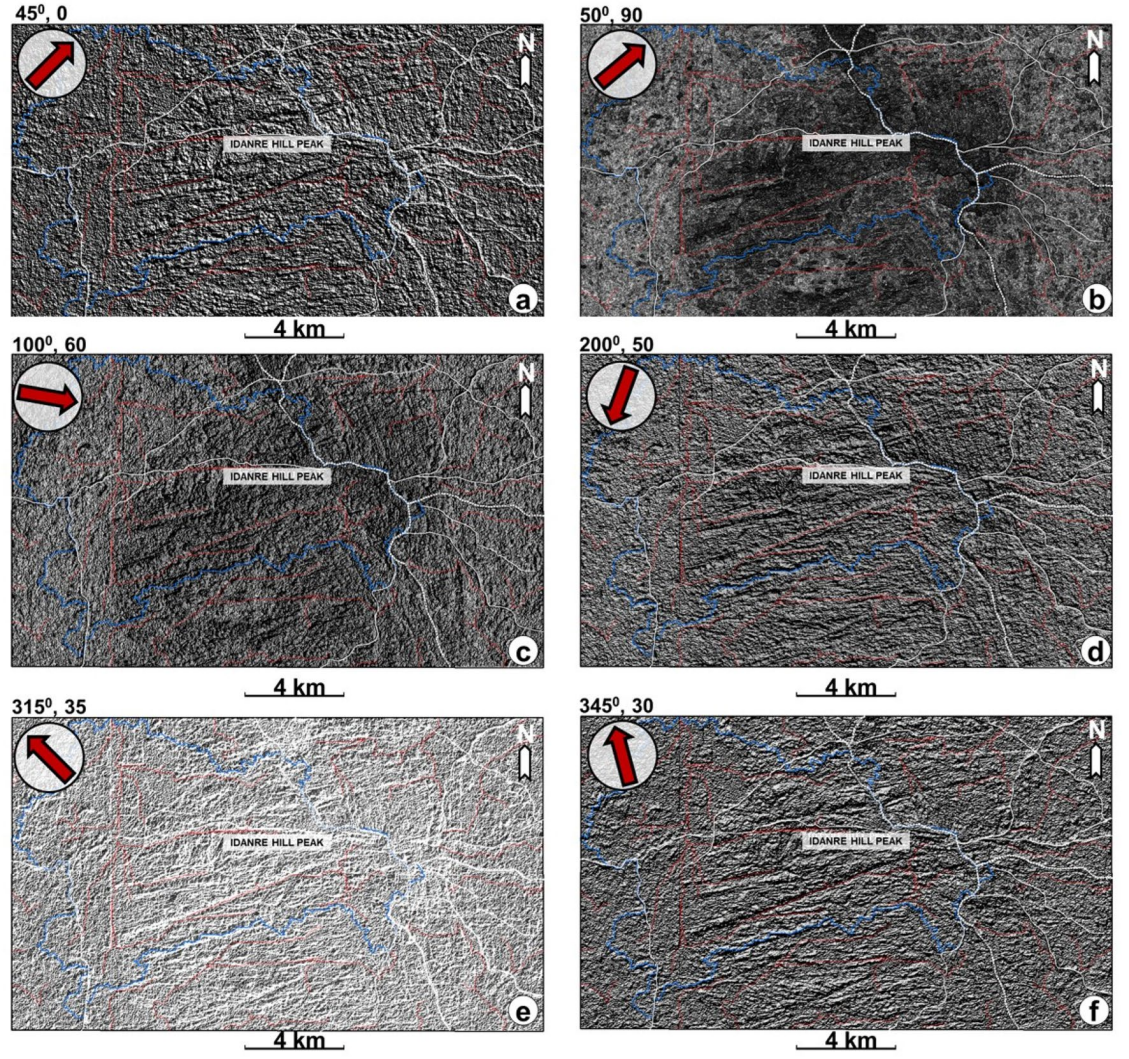
Multi-directional hillshade maps overlain by stream network (red lines) and watershed (blue lines) of the study area. The hillshade maps are generated from google earth digital surface model (DSM) using different azimuth angles shown by the inset red arrows. (**a**) 45° azimuth (**b**) 50° azimuth (**c**) 100° azimuth angle (**d**) 200° azimuth angle (**e**) 315° azimuth and (**f**) 345° azimuth.

**Figure 6:**
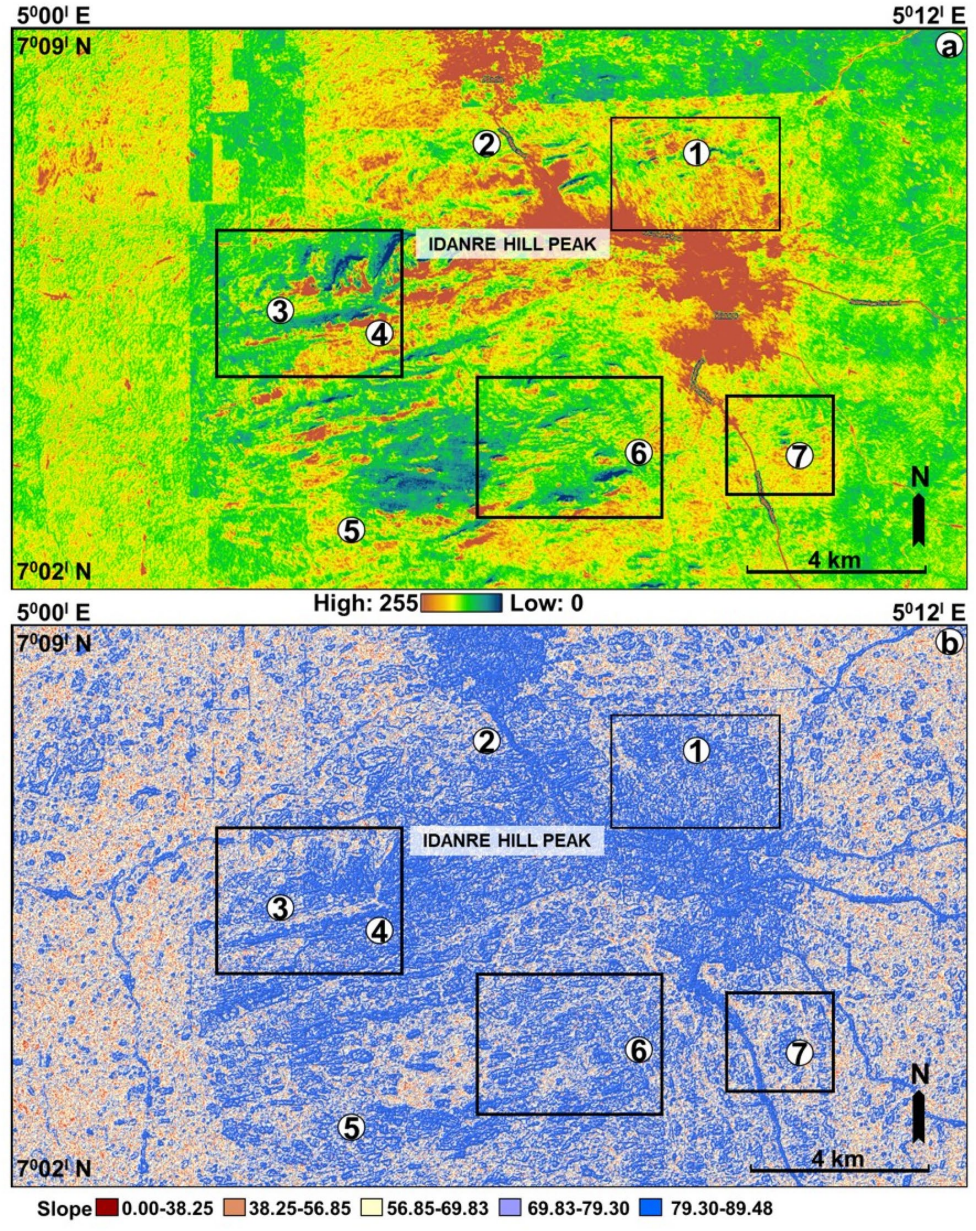
(**a**) Elevation map of the study area as rasterized from the google earth DSM in Fig. 2a. (**b**) Slope attribute as derived from the spatial analysis tool in ArcGIS. The map is used to identify changes in slope (gradient or steepness) within the study area.

The study area is drained by E-, W-, N-, and S-flowing streams (Figs. 4 and 5). The N-flowing streams are common in the western and northern parts, while the E- and W-flowing streams occur mainly in the southern, central, and eastern parts of the study area (Fig. 4a). The streams in the eastern part exhibit a radial drainage pattern around the high and low elevation areas i.e., in areas coincident with rock exposures outcrops, and the Idanre township (Fig. 4a,b). The N- and W-flowing streams are connected in the west, where they collectively exhibit dendritic drainage pattern and form the largest drainage and most interlinked network of streams within the study area (Fig. 4). The delineated watershed covers approximately 86 km^2^ in area and extends from the central to the western part of the study area, where it bounds several N–S and E–W oriented streams (Fig. 4a,b). Topographically, the center of the study area is characterized by intermediate to high elevation regions, with sparsely-distributed low elevation areas mostly surrounding the high elevation spots (Figs. 4a and 6a). The same areas are characterized by intermediate to predominantly high slopes (Fig. 6b). The western and eastern parts of the study area are associated with intermediate elevation values, except at sparsely distributed outcrop locations, such as in the northeastern axis where elevations are locally high (Figs. 4a and 6a). Slope values in these areas are generally low. However, high slope, albeit sparsely distributed, are observed at outcrop sites in these areas (Fig. 6b). The northern half of the study area is more broadly characterized by continuous patches of orange color, indicating high elevation values within areas that are linked to settlements (Fig. 6a). These areas are also associated with high slope values and are flanked by high elevation outcrop sites from which the streams emerge, reaching low elevation and low slope areas in the east and west, respectively. In the west, these areas fall within the watershed and form the topographic lows for northward flowing streams (Fig. 4).

### Lineament orientation and distribution

**Figure 7:**
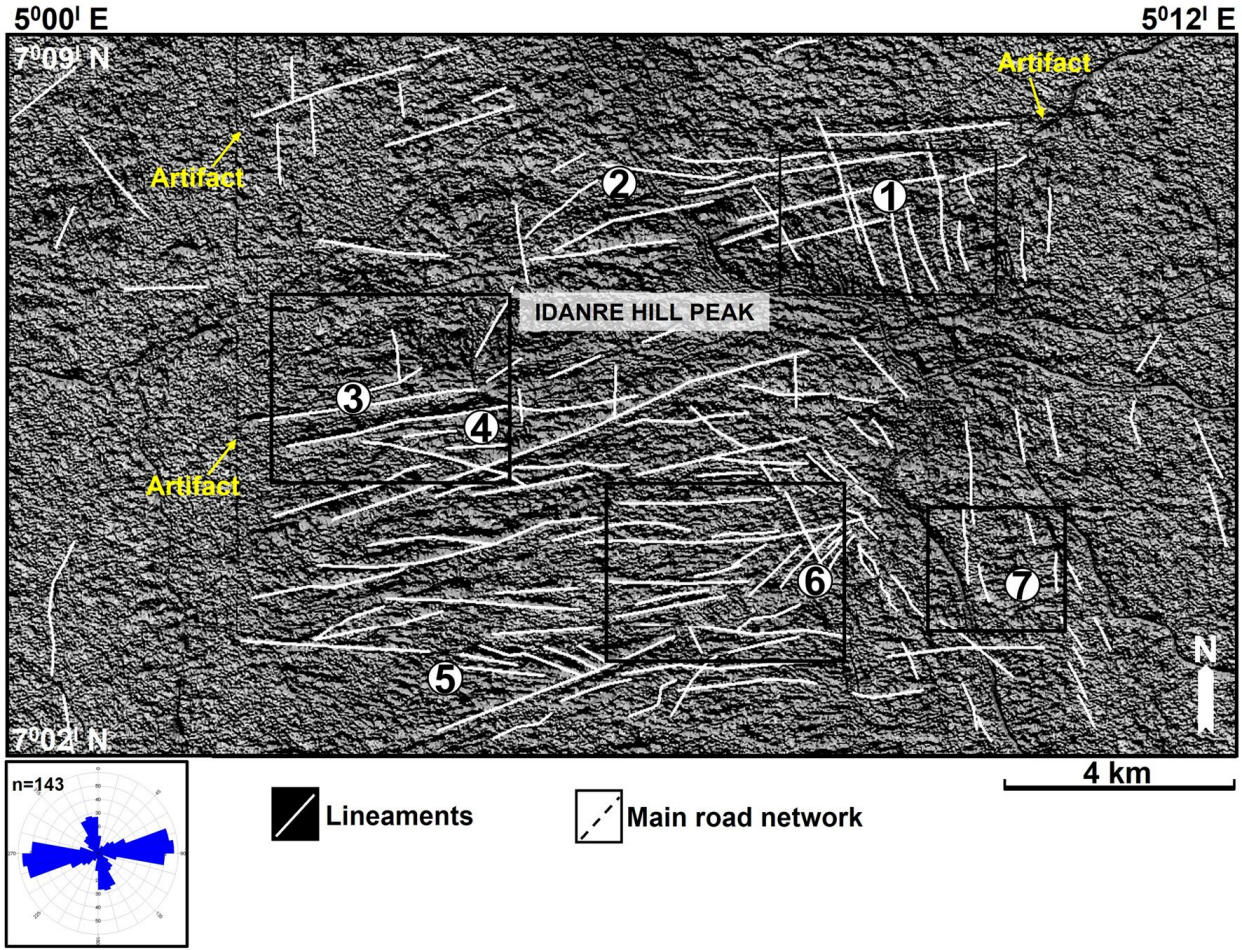
Distribution of lineaments within the study area. The lineaments are extracted from the hillshade maps in Fig. 4 and are here displayed on the 200° azimuth angle hillshade map. N.B: Rose diagram shows the strike of the lineaments.

**Figure 8:**
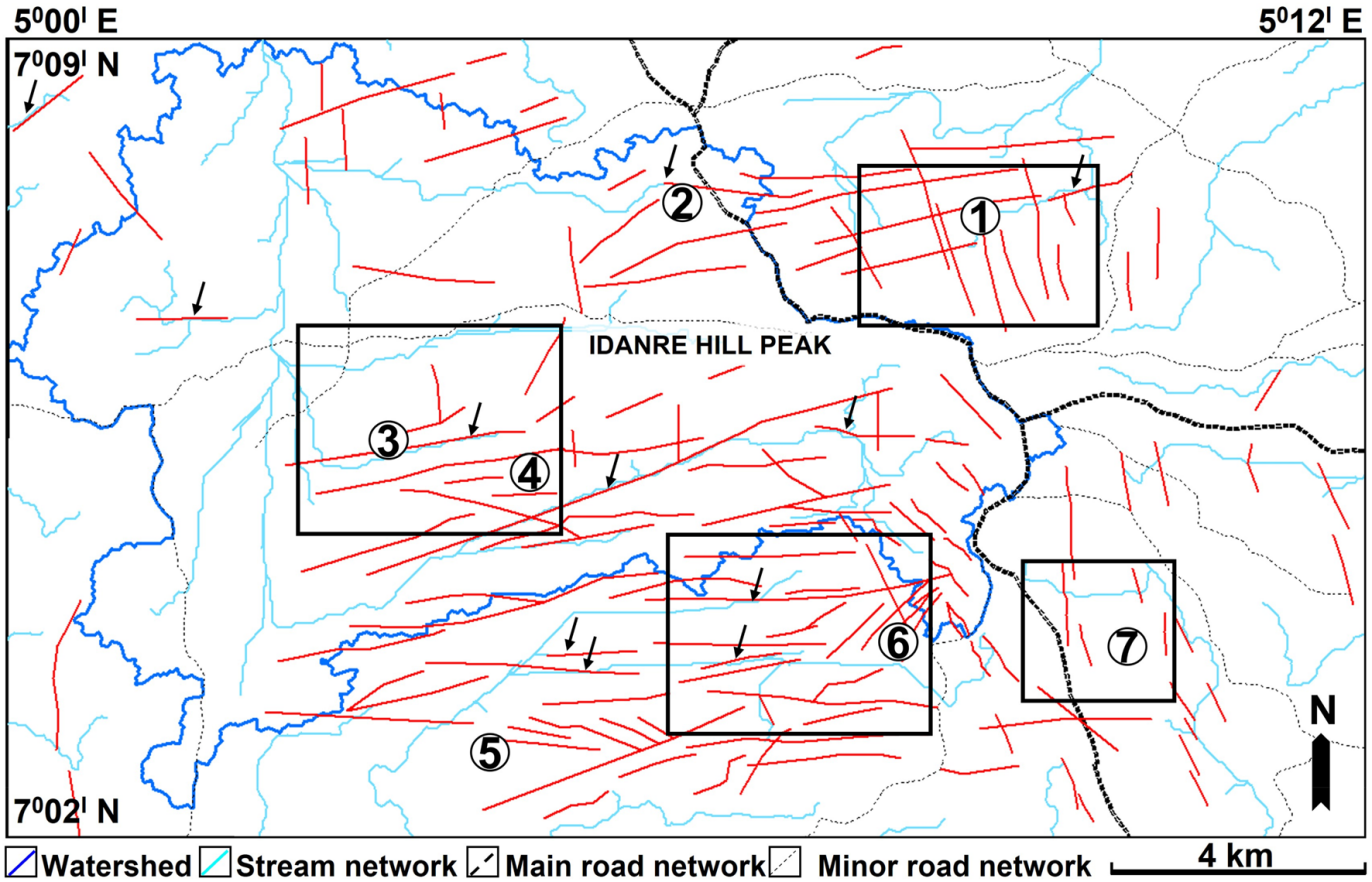
Stream network overlain on extracted lineaments within the study area. Black arrow shows areas where a stream segment overlaps with a lineament.

143 lineaments were extracted from the six multi-directional hillshade maps in Fig. 5a–f. Analysis of these lineaments and rose plot reveal the occurrence of ENE-WSW, NNW-SSE, N-S, NW–SE, ESE-WNW, E-W, and NE-SW-trending lineament populations, with the ENE-WSW trend being predominant (Figs. 5, 7, and 8). The northern half of the study area is dominated by NNW-SSE and ENE-WSW oriented lineaments that are widely observed in the north central part, where points 1 and 2 are located (Figs. 7 and 8). Minor occurrences of N-S, ESE-WNW, E-W, and NE-SW oriented lineaments are recorded outside the north central area (Fig. 5). The southern half is dominated by ENE-WSW oriented lineaments, followed by the NNW–SSE oriented types. This is in addition to minor N–S, NW–SE, and WNW–ESE oriented lineaments that occur in the area (Fig. 7). Within this area, lineaments are highly concentrated in the south-central part. On the other hand, their distribution in the southwestern part of the area is highly-sparse, as only two lineaments (NNW–SSE and NNE–SSW trending types) were identified there. In addition, lineaments are more abundantly distributed in the eastern part, which is dominated by the NNW-SSE trending types (Fig. 7).

**Figure 9:**
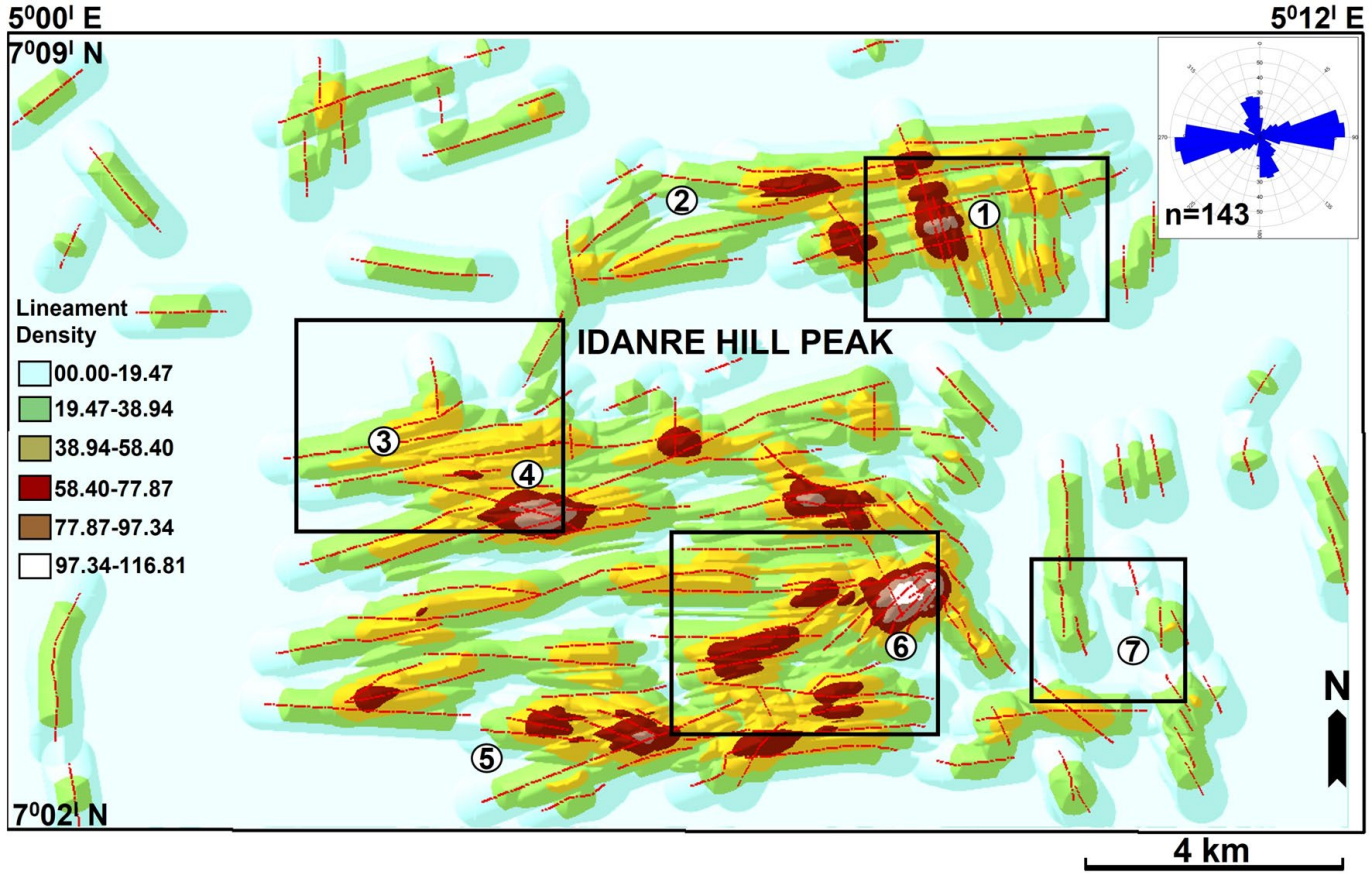
Lineament density (/km^2^) map of the study area as derived from the digitized lineaments from the hillshade maps in Fig. 4. The rose diagram is used to demonstrate the strike of the lineaments.

In terms of their interactions, lineaments show marked geometrical and topological relationships (Figs. 7, 8, 9). Geometrical relationships between the lineaments include isolated, abutting, cutting, and mutually cutting relationships. All the types of fracture topological relationships proposed by^54^ such as I-, X-, and Y-nodes are also observed (Fig. 7). For example, the NNW-SSE and ENE-WSW lineaments intersect at multiple points close to point 1 where they exhibit mutually intersecting geometries. Three NW–SE oriented lineaments abut on a NE–SW oriented lineament to the west of point 5 (Fig. 7). This relationship is also observed among lineaments in points 4 and 6, and south of point 7. With respect to streams and other landmark features, some lineaments in the study area overlap with stream segments. This relationship is observed near points 3 and 4, where the traces of two ENE–WSW oriented lineaments correspond to segments of two similarly oriented streams (black arrow in Fig. 8). This character, although not prevalent, is also observed northward of point 6, west of point 6, and northeast of point 5 (Fig. 8). Similarly, some lineaments are observed near or within settlement areas, where they extend across roads and buildings within the Idanre township (Fig. 8).

### Lineament density mapping

Computed lineament density ranges from 0.00 to 116.81 per km^2^ (Fig. 9). Areas with high lineament density (97.34–116.81) are predominant in the center of the study area, while areas with low to zero (0.00–19.47) lineament density are widespread in zones surrounding the lineaments, and in the eastern and western parts of the study area (Fig. 9). High lineament density values observed in the center of the study area are associated with a NE-SW trend, which appears as though the lineament density patterns collectively reflect an underlying highly deformed or anomalous geological feature (Fig. 9). In the northern half of the study area, the highest lineament density is observed at lineament intersection points close to point 1. This is followed by high lineament values in areas in-between points 1 and 2. In the southern half, high lineament density values are observed near point 4, east of point 5, and north and west of point 6 (Fig. 9). These zones are associated with lineament intersections and abutment as shown in Fig. 8. The highest lineament density is associated with point 6.

### Terrain roughness assessment

**Figure 10:**
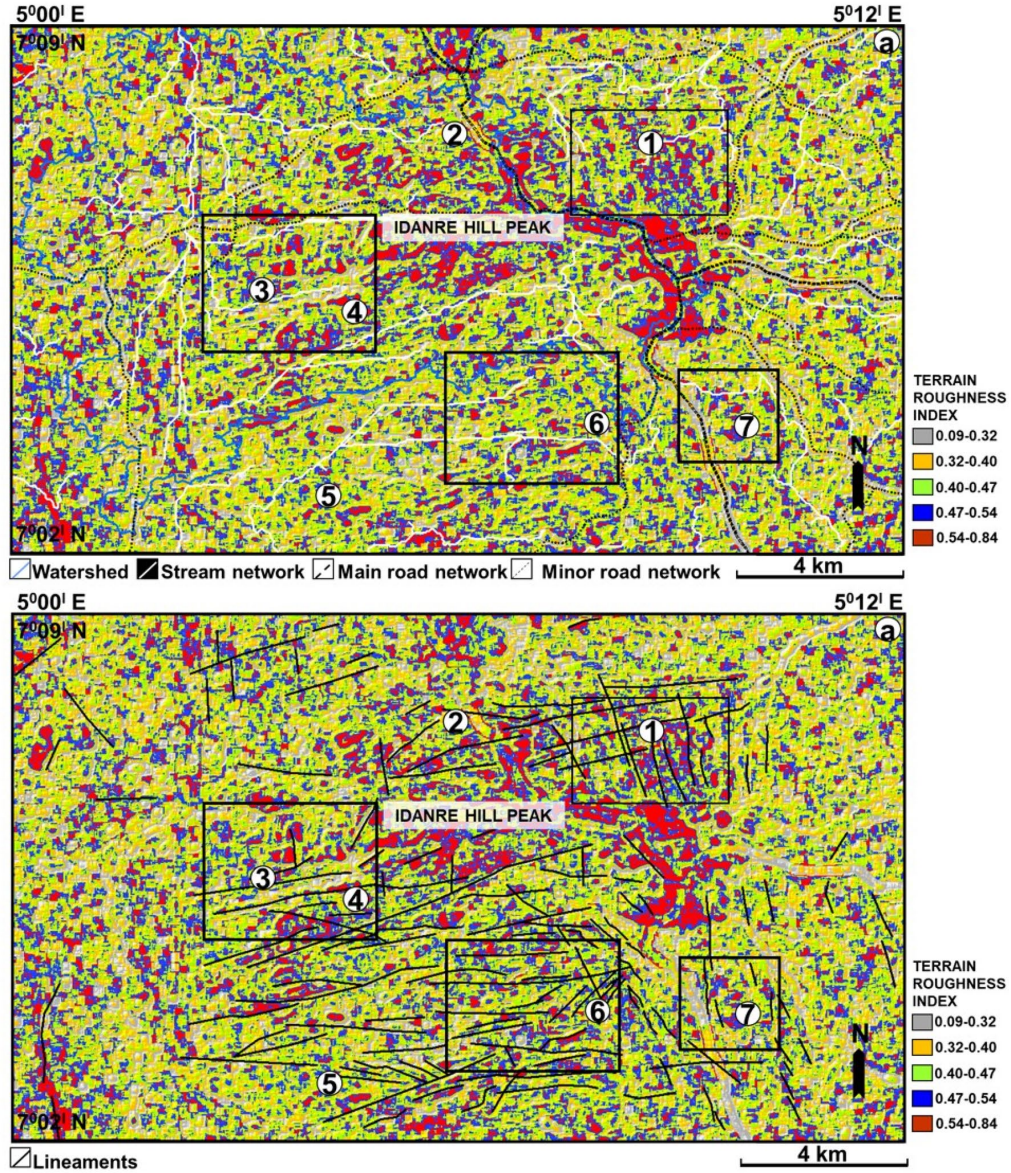
Terrain roughness map showing changes in terrain properties especially at points 1–7. (**a**) with stream network and road overlain and (**b**) with lineaments overlain.

Computed terrain roughness index ranges from 0.09 to 0.84 (Fig. 10a,b). The largest concentration of high roughness values is observed as a continuous NW–SE trending red patch in the settlement areas. In addition, high roughness values are distributed in major parts of the study area and are particularly observed at the sites of the lineaments and outcrops in the center of the study area and in few other parts of the study area (Fig. 10b). For example, in the southwestern part, the two lineaments with NNW-SSE and NNE-SSW trends are associated with red patches indicating high terrain roughness values at these locations (Fig. 10b). A similar relationship is observed close to points 3 and 4, where outcrops and lineaments are present (compare Figs. 2 and 8).

### Validation of structures with google earth imagery

**Figure 11:**
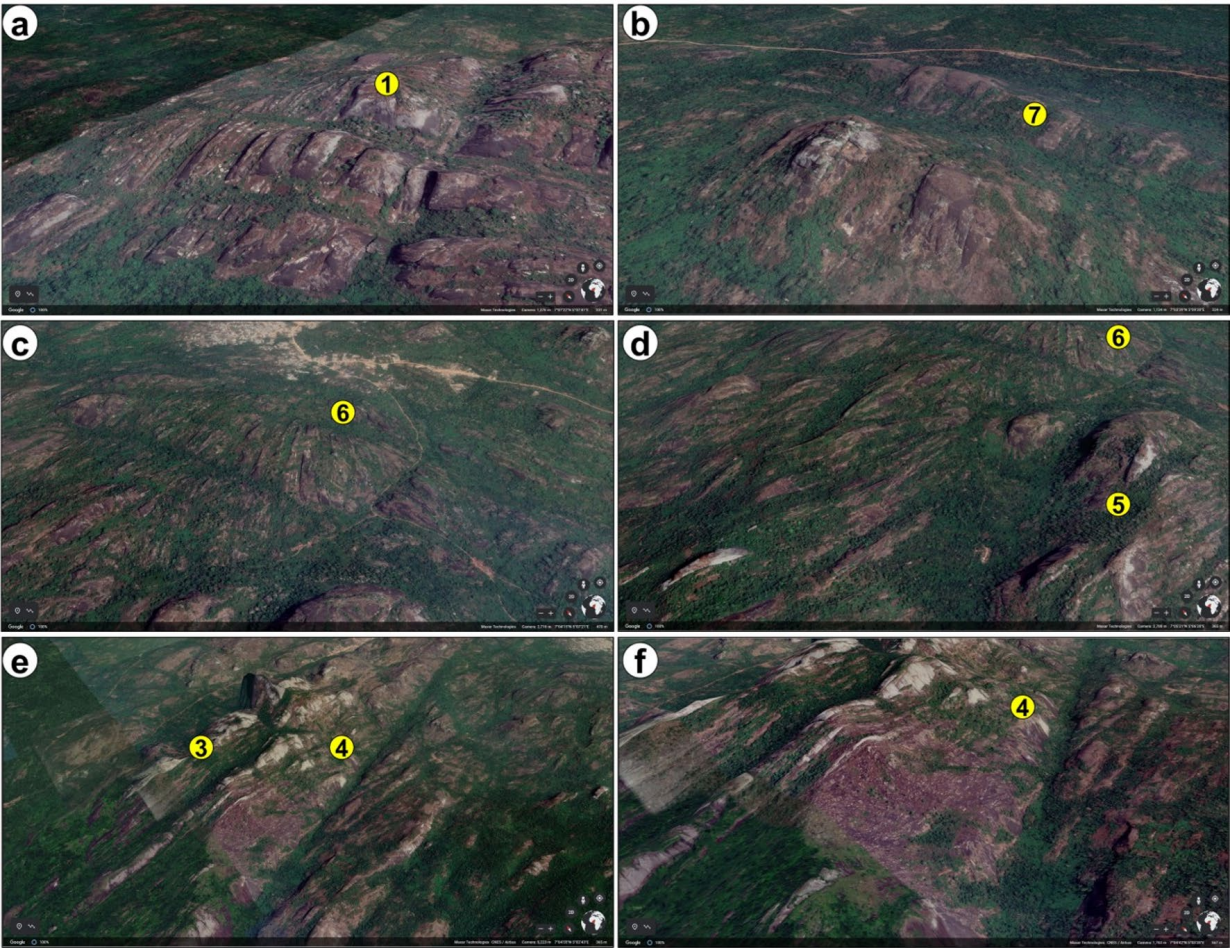
Uninterpreted and virtually realized 3D images of points 1–7. Maps data: Google Earth, Maxar Technologies and CNES/Airbus.

**Figure 12:**
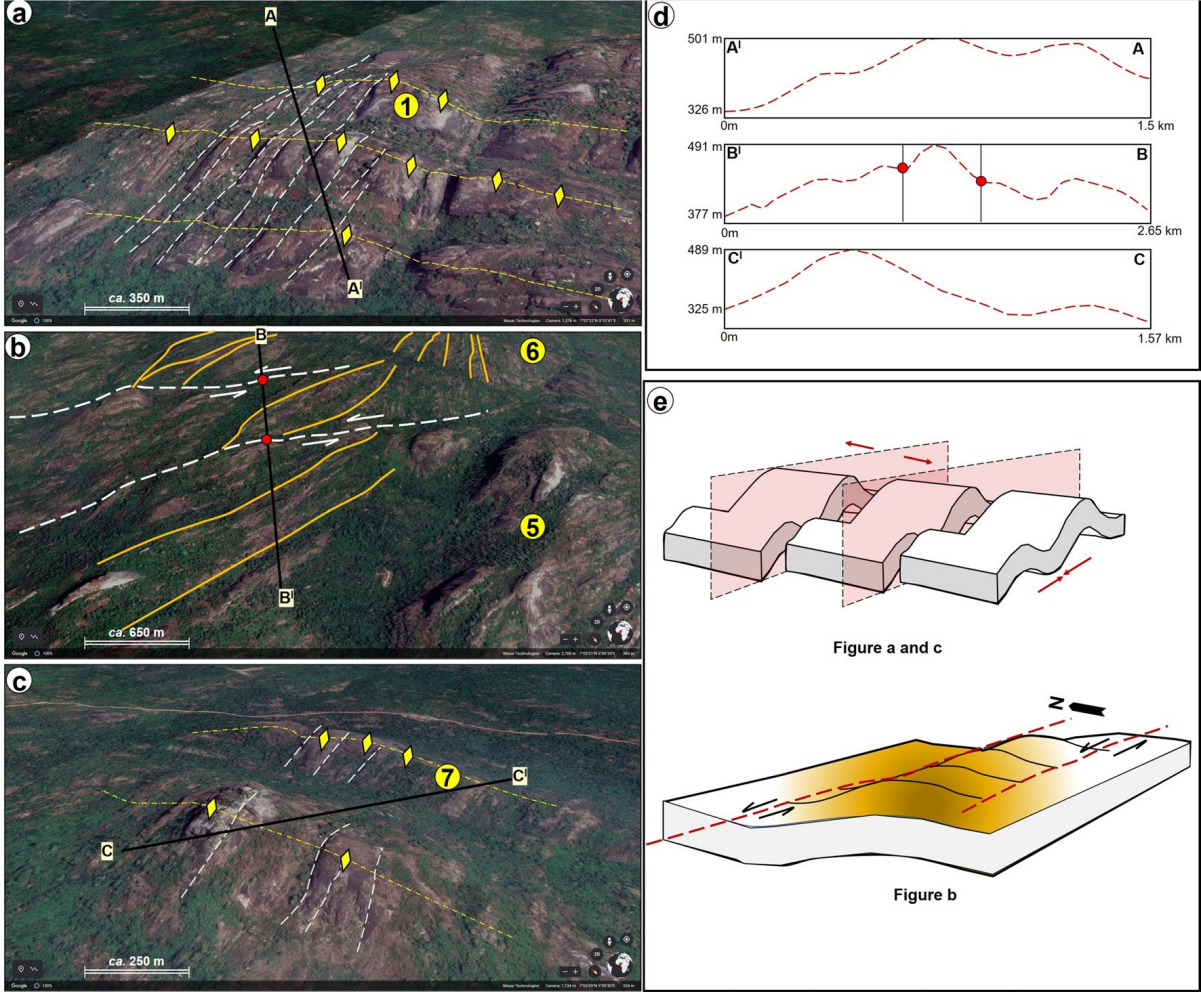
(**a**) Interpretation of structures from some of the selected points (**d**) Transects and topographic profiles the structures indicated in (**a**)–(**c**) and (**e**) schematic structural model for the interpreted images in (**a**)–(**c**). Maps data: Google Earth, Maxar Technologies and CNES/Airbus.

Given the high spatial resolution of google earth aerial photographs, it is of interest to understand to what extent observations from the generated maps correspond to real life terrain features on google earth images. The study area comprises km-scale massive high elevation outcrops with steep sides and an undulating topography i.e., rock blocks with sharply changing elevation (Figs. 11a–f and 12a–d). These rocks are dome-like, and they locally constitute several extensive rock chains that are separated from one another by vegetation and low-lying areas. The dome-like blocks exhibit a NW–SE trending axis (Fig. 12a,c). Relative to lineaments, point 1 which is associated with high lineament density in Fig. 9 is linked to a series of ENE-WSW trending linear features or lineaments that separate the deformed rock mass into several folded blocks (Figs. 11a and 12a). The tip of the folds as mapped on a transect across three ridges in the area, can reach up to 501 m in height (Fig. 12a,d). This is corroborated by Fig. 1 which reveals high elevation values of up to 900 m at the Idanre Hills peaks as against the 100 m height in surrounding areas. Several NE–SW trending linear features are also observed near point 6 (Figs. 11c and 12b). These features are like those observed in Figs. 5 and 6, which confirms that the area is a very high lineament density area (Fig. 9). Outcropping rocks seen in the central part of the study area (Fig. 2a,b) are also observed to have sharp changes in elevation i.e., undulations, and white–grey patches (Figs. 11 and 12), which appear to be related to the intermediate to high terrain roughness values observed in the lineament and outcrop sites (Fig. 10).

## Discussion

This work demonstrates the effectiveness of using GIS techniques and google earth aerial photographs to map and provide preliminary data on lineaments. Since this work provides a reconnaissance tool, there is no ground truth data. Hence, no rigorous interpretation of the geological features (at outcrop scale or less) is provided. Nevertheless, the approach and results from this work have overarching applications for.

### Lineament characterization

The integrated approach used here revealed structures with different orientations and those that were probably hidden by the vegetation cover. The application of multi-directional hillshade maps proved to be reliable in mapping lineaments within the study area in a similar way as previously done in other terrains such as in India’s Pravara basin^21^ and in the north and southeastern desert of Egypt^8^. The 143 lineaments extracted in the study area are distributed at several outcrop sites in the center of the study area. The lineaments have variable orientations, including NNW–SSE, ENE–WSW, NNE–SSW, ESE–WNW, E–W, SE–NW, and NE–SW trends, with the ENE–WSW trend being the most dominant. This observation is in line with previous studies, which relied on the analysis of Landsat Enhanced Thematic Mapper Plus and Advanced spaceborne thermal emission and Reflection Radiometer (ASTER) DEM data covering the Idanre Hills and surrounding areas^17,36^. Thus, reflecting the power of google earth imagery and its derivatives in extracting meaningful geological information especially in the absence of ground geological field work.

The lineaments mapped in this study generally represent zones of weakness or fractures, which formed following several phases of local or regional deformation. Their orientations suggest they can be classified as similar features identified previously in the study area. The NNW–SSW/NNE–SSW and ENE–WSW-trending lineaments may correspond to similarly oriented fractures mapped on field and satellite data, which are oriented parallel and normal to the long axis of the Idanre batholith, respectively^36^. Additionally, the NNW-SSW and NNE-–SSW fractures are likely to have fostered the emplacement of the Idanre batholith forcefully within the surrounding migmatite-gneiss^53^. On the other hand, the ENE–WSW fractures may evidence slow cooling at depth and shrinkage due to compressive force that accompanied the emplacement of the batholith^36^. On a regional scale, similar NNW–SSE and ENE–WSW-trending fractures have been observed from other areas within the basement complex of Nigeria e.g.^17^. Hence, signifying that these lineaments may be linked to regional episodes of deformation or polycyclic tectonic history that characterizes rocks within the basement complex of Nigeria^47,55^.

### Lineament density: variability and controls

Analysis of lineament density reveal variable lineament density values across the study area (Fig. 9). Areas with high density are associated with the occurrence of outcrops, intersecting to abutting lineaments, and high lineament clustering. This indicates high degree of lineament connectivity^56^. The behavior is manifested in points 1 and 6 where high lineament cluster and density are revealed by intersection and abutting relationships between lineaments, and a moderately to highly rugged terrain (Figs. 6, 7, 8, 9 and 10). Several deformation-related features which indicate the occurrence of high strain domain that may enhance fracture density are also identified in the vicinity of point 6. These are variably oriented linear features which deform folded or dome-like, high elevation, rock blocks across the center of the study area (Figs. 11 and 12a–c). On the contrary, intermediate to low lineament density values observed in the eastern and western sections of the study area are due to the paucity of outcrops there (Figs. 2a and 9). Hence, areas with high lineament density are likely to be the most deformed, while areas with low lineament density are expected to have lower degree of deformation. Similar hypothesis was made by^57^ in the Suoimuoi catchment, northwest Vietnam based on the distribution of lineaments there.

Consequently, the variable lineament density and lineament distribution patterns observed in the study area may be due to lithological or structural controls^58^. The preferential clustering of the fractures in the center of the study area as confirmed on the lineament density map indicates that the center was more evidently deformed than surrounding areas i.e., a high strain domain or that the rocks in the central area/core were more sensitive to brittle deformation. Reference^59^ suggest that enhanced straining will cause increased fracture intensity. The center of the study area, where outcropping rocks were identified corresponds to exposed sections of the Idanre batholith, which has been suggested to be better deformed more than the surrounding migmatite country rock^36^. This deformation likely represents imprints of emplacement tectonics, which generally predisposes the granitic outcrops in the central part of the study area to higher degree of deformation unlike the surrounding areas where our observation suggests lower degree of deformation. Our observations suggest that the variable lineament density and lineament distribution in the study area are dictated by the rock type and degree of deformation. In addition, the abundance and paucity of outcrops in the central and other parts of the study area, respectively corresponds to the distribution of lineaments in these areas. Hence, the distribution of lineaments in the Idanre Hills area is primarily controlled by the spatial distribution of rocks/outcrops.

### Terrain roughness versus rock weathering and fracture behavior

Our results further indicate that south of point 1 and north of point 2, the high terrain roughness values are due to the presence of settlements/houses (Figs. 2 and 10). These high values may signify the level of surface complexity or topographic texture associated with the settlements^19^. In addition, high terrain roughness values observed in outcrop areas may correspond to the white–grey patches and fractures observed on the google earth images in Figs. 10, 11 and 12. High terrain roughness in outcrop sites can reflect moderate to high weathering of the rocks since differential rock weathering commonly leads to increased surface roughness^60^. Evidences of physical, chemical, and biological weathering, including disintegrated coarse rock boulders, mineral alteration, and rock surfaces habited by plants were documented on rocks within the Idanre Hills area^53^. This widespread activity presumably jointly contributed to the high terrain roughness values observed in the study. In addition, the high terrain roughness observed at lineament sites (Figs. 2 and 10b) indicates an abundance of fractures of elevated roughness, which can enhance rock weathering and that these fractures greatly enhanced the high roughness values observed in the study area.

### Lineament-influenced streams within the Idanre Hills area

Results of stream network analysis reveals that several streams are distributed around the high elevation areas (Fig. 4), showing a dominant radial drainage system albeit dendritic to the west. These streams are interpreted to flow in the north (mostly), east, west and south direction. They commonly flow away from the high elevation outcrops and residential areas before eventually flowing westward and eastward, where slope and elevation generally decrease (Fig. 4). In the west, they flow into low elevation areas in the watershed, where the flow is then re-routed northward through N–S oriented streams. This observation coincides with trends from previous studies which show that major rivers in the Idanre Hills area exhibit a general N–S orientation of flow, which is parallel to the foliation observed via field studies in the migmatite-gneisses of the Idanre Hills^36^. Accordingly, some stream segments near points 3 and 4 coincide with the ENE–WSW oriented lineaments (Fig. 8). Although this observation is not prevalent in the study area, it nevertheless shows the impact of lineaments in influencing drainage locally within the area. It also strengthens previous studies, which reveal that certain ENE–WSW oriented lineaments are sources of numerous springs, streams, and rivers within the Idanre Hills and surrounding areas^36^. The occurrence of lineament-influenced streams is not exclusive to the Idanre Hills area as similar relationships have been identified in other areas such as the Béré region of Ivory Coast^61^ and India’s Pravara basin^21^.

### Conceptual structural models

Observations from google earth images, although are not totally differentiated, suggest that the study area is deformed by lineaments, which may correspond to deeply buried, and through-going fractures. The lineaments are interpreted to be associated with other deformation features such as folds (domes) and shear zones (Fig. 12a–c,e). The structural models in Fig. 12e show that the rock masses have undergone various types of deformation, including compression, evidenced by folding and the dome-shaped morphologies. These rocks were likely folded because of any or combination of the following (a) folding in response to a dominant ENE-WSW compression, resulting in NNW-SSE fold axes trends (upper model in Fig. 12e) and (b) large-scale shear tectonics associated with the last phases of the Pan-African orogeny. In a latter event, the folded rock masses were intersected by ENE-WSW oriented lineaments following a main NNW-SSE extension (Fig. 12a,c,e). Consequently, the rock blocks close to points 5 and 6 locally appear to have moved relative to one another (Fig. 12b). Here, we interpret this trend to reflect left-lateral (sinistral) type strike-slip faulting or shear motion and deformation of the rocks bounded by these lineaments as this appears to be a plausible explanation for the observed behavior in the absence of ground truth.

### Caveats in the interpretation of lineaments and terrain attributes

This study highlights the effectiveness of integrating GIS techniques and google earth imagery for lineaments and terrain attributes mapping within the Idanre Hills area. However, it is important to mention the downsides of generating slope, elevation and stream networks of an area in the vicinity of settlements from rasterized google earth DSMs. For example, despite identifying settlements in low elevation and low slope portions surrounding the Idanre Hills peaks on google earth images, these same settlements are associated with high slope and high elevation values on our computed slope and elevation maps, respectively. Expectedly, low-lying areas would generally have low elevation and low slope when compared to the surrounding Idanre Hills, where outcrops are up to 900 m high. This observation reveals that the slope and elevation values derived for the settlement areas were most probably computed based on the analysis of the texture of the rasterized google earth DSM. This may as well have an impact on the terrain roughness values generated in the settlement areas.

Regarding the distribution of streams within low-lying areas such as where the settlements are located, we identified that the streams, which were automatically generated are distributed outwards away from the settlements. This is likely because the drainage algorithm recognized the settlements as high elevation and high slope areas (Fig. 7a,b). Therefore, care must be taken while using google earth DSM to automatically extract drainage network in areas close to settlements. Similarly, stripes i.e., artefacts were identified in the western side of Fig. 2a. These continuous stripes are acquisition footprints resulting from overlap of the aerial photographs, e.g.,^62,63^ and they were replicated on all the generated maps, including in Figs. 4a, 5 (Hillshades), 6 (elevation and slope), and 7 (Hillshade). We suggest that the impacts of the stripes on lineament and terrain mapping are minimal as they are restricted to a limited portion of the data in the western part of the study area.

## Conclusions

The approach used in this study is recommended for remote mapping of geological features in inaccessible locations, for initial-stage or first step geological field studies, and for planning prior to detailed field mapping and project execution. It is also recommended as a simple teaching guide to help students easily understand the distribution and behavior of lineaments and terrain characteristics within a given area.

The conclusions from this study are that:

- Lineaments distribution, density and trend vary across the study area.
- Lineament distribution and density are dependent on the underlying rock types.
- Outcrops and lineaments are commonly associated with high terrain roughness values, indicating that lineaments with elevated surface roughness are widespread in the study area.
- Drainage in the study area is partly lineament-controlled.
- Google earth DSM should not be used in isolation but should be combined with other satellite data such as SRTM and ASTER DEM for more reliable remote mapping of geological features and importantly, terrain (e.g., slope, elevation and roughness) prior to ground truth.

### Methods and workflow

The method used in this work is divided into two main parts (a) Data collection and (b) Data analysis.

### Data collection, quality control and preparation

The data analysis part includes the download of DEM data from any open source GIS platform like Topex, GEBCO, USGS Explorer, OPENDEM, and Google Earth. In this study, the DEM of the study area, with a spatial resolution of 15 arc seconds*,* was download first from GEBCO (www.gebco.net/) and gridded in golden software Surfer 16 (Fig. 1a). Satellite or aerial photographs of the study area were later captured from google earth (https://earth.google.com/web/), georeferenced, and exported as joint photographic expert group (JPEG) file (Fig. 2). The google earth images, obtained on 15-11-2021, have a spatial resolution of 4–10 m, which is enough to conduct regional geological studies. The JPEG file was uploaded into Esri’s ArcGIS 10.7.1 ArcMap prior to data analyses. To use the imported aerial photograph, it was first georeferenced in the correct coordinate projection in ArcMap i.e., Minna/Nigeria West Belt (EPSG:26391 with transformation: 1168). Rasterization of the aerial photo was done in ArcGIS through the sub-processes shown in Fig. 3. The rasterization process involves conversion of the vector graphics format (JPEG) of the google earth image into a raster image (pixels or dots). An important aspect of the rasterization process is to update default setting in the environment tab in ArcMap to allow for optimum selection of the best resolution, storage, and cell size for the rasterized image. The google earth-derived image was the DSM on which further GIS analysis was performed using different tools available in ArcMap.

### Data analysis

The data analysis is sub-divided into four parts (a) generation of stream network (b) lineaments mapping (c) terrain roughness index calculation, and (d) extraction of structural information from focused and high resolution google earth imageries (Fig. 3).

#### Generation of stream network

The stream network algorithm was built in the Spatial Analyst Tool (SAT) box in ArcMap (Fig. 3). First, the rasterized map was filled in ArcGIS using the Fill command, which fills sinks in a surface raster to remove small imperfections in the rasterized data^64,65^. A sink represents a cell with an undefined drainage direction. Afterwards, the flow direction and flow accumulation raster calculations were executed (Fig. 3) to (a) create a raster of flow direction from each cell to its downslope neighbor, or neighbors, using D8, Multiple Flow Direction (MFD) or D-Infinity (DINF) methods^66^ and (b) to create a raster of accumulated flow into each cell. The flow accumulation tool calculates accumulated flow as the accumulated weight of all cells flowing into each downslope cell in the output raster^67^. The calculated Flow accumulation map was further classified into two groups of 0 to 5000 and 5001–371,000. Then, the map algebra was used to modify the final map as Flow accumulation of ≫ 5000 i.e., the stream data. Subsequently, the watershed was delineated following the Basin command, which determines the contributing area above a set of cells in a raster and allowed all the drainage basins in the study area to be mapped (Fig. 3). To observe the influence or control of lineaments on the stream distribution and flow in the study area, the extracted stream network was overlain on the lineament map. This is important to assess areas where water or streams flow are influenced by fractures or cracks^68^.

#### Lineament extraction

To accurately identify lineaments in the study area, several topographic attributes such as Hillshade, Slope, Curvature, and Aspect were calculated (Figs. 3, 4, 5 and 6). The Hillshade attribute creates a shaded relief from a surface raster by considering the illumination source angle and shadows^69^. The aspect is derived from a raster surface and is useful for identifying the downslope direction of the maximum rate of change in value from each cell to its neighbors^70^. This is a useful attribute for validating stream trends from lineaments. In addition to the hillshade and aspect, the slope attribute was calculated to identify the gradient or rate of maximum change in z-value from each cell of the rasterized map. To optimize mapping of the lineaments, subsets of the derived hillshade map were generated as a function of azimuth and elevation. For this work, the light illumination azimuth angles of the shaded relief maps are 45°, 50°, 100°, 200°, 315°, and 345° respectively (Fig. 4). Furthermore, the lineaments were digitized on each hillshade manually following the approach of^71^. As linear cultural features such as roads and streams are not of interest here, we ensured they were not picked as lineaments by carefully picking only linear features which are geology-related. Moreover, the digitized stream network, watershed and roads were frequently compared to the suspected lineaments before the latter were digitized. Once the lineaments were digitized on all the hillshade maps, the extracted lineaments were again validated against the trend of streams, major and minor roads, and artifacts produced from the original rasterized map. Lineament density was subsequently calculated under the SAT following the density command and by choosing the option of ‘line density’. Importantly, the default parameters were changed to cover the study area alone. The lineament density (L_d_) describes a 2-D concentration of lineaments within a given space and computed by dividing the total length of lineaments (Ll) by the area (A) under consideration^72^. The lineament density was calculated in square kilometers, while analyses of strike or trend of the lineament was done in Rockwork software.

#### Generation of terrain roughness

The Terrain Roughness Index (TRI) estimates the amount of elevation difference between adjacent cells of a DEM^73^. The TRI calculates the difference in elevation values from a center cell and the eight cells immediately surrounding it. Then it squares each of the eight elevation difference values to make them all positive, sums them, and takes the square root^73,74^. The TRI map is applicable for the characterization of geological terrains, modelling sediment transport, ecological studies, geomorphological evaluation of landforms, and landslide hazards assessment^73,75–78^. In the study area, the TRI was computed in two steps (Fig. 3). The Min (minimum), Max (Maximum) and Ave (Average) differences in elevation were first estimated using the ‘Neighborhood’ and ‘Focal statistics’ tools under SAT (Fig. 3). In a second step, the TRI was calculated by dividing the difference between the Ave and the Min by the difference between the Max and the Min (Fig. 3).

#### Extraction of Google Earth Images for structural mapping and features validation

The last step in the workflow is the extraction of geological and structural information from the Google earth imageries (Fig. 3). The five areas with prominent or striking structures observed in Fig. 2 were studied in both plan and 3-D view. In this step, the grids were defined over the study area in Google Earth pro. The five locations around the Idanre Hills were defined as Loc 1 to Loc 5 and saved as KML for subsequent use in Golden software Surfer 16. The coordinates of the defined grid were used to extract topographic information from both Topex (https://topex.ucsd.edu/cgi-bin/get_data.cgi) and in GEBCO (www.gebco.net/). The topographic data from both sources were gridded in Surfer 16 using the Kriging method. The final elevation map was based on the Topex data since it provided more resolution of the topography. The elevation information was used for making selected topographic profiles across the 5 areas of interest. Pulsating structures from the five locations were further analyzed for their attitude in google earth, which formed the basis for the structural models in Fig. 12. Resulting images were also used to validate results of terrain analysis in the study area.

## Data Availability

The images used in this study are available upon request and can be downloaded online using Google earth tools: https://earth.google.com/web/. Elevation data can be downloaded from GEBCO: https://download.gebco.net/ and Topex global topographic data: https://topex.ucsd.edu/cgi-bin/get_data.cgi.
